# Machine Learning Quantifies Fine‐Scale Hairiness in Shore Flies (Diptera: Ephydridae)

**DOI:** 10.1002/jmor.70096

**Published:** 2025-10-14

**Authors:** Shawn M. Abraham, Marcos Rodriguez, Victoria Hristova, Felix A. H. Sperling

**Affiliations:** ^1^ Department of Biological Sciences University of Alberta Edmonton Alberta Canada

**Keywords:** adaptation, electron microscopy, microtrichia, pixel classification, semiaquatic

## Abstract

Morphological analysis of fine structures on small insects is often labor intensive, scale‐limited, and biased by sampling or organismal life history. We used a pixel classification machine‐learning workflow with the open source programs Ilastik and Fiji to identify and quantify microtrichia in semiaquatic shore flies (Ephydridae). This methodology semi‐automates quantification of hairs by counting objects or groups of class‐assigned pixels and determining their percent coverage at a given magnification using scanning electron micrographs. Our results are consistent with manual counts, with *Paracoenia* species that tolerate hot springs having more hairs than less aquatic *Parydra*. However, *Paracoenia* hairs tend to be shorter, and the percent coverage of microtrichia per unit surface area did not differentiate species except for the anterior thoracic spiracle. Our workflow is adaptable for use in other taxonomic groups or beyond the quantification of hairs, with the upper limits of applicability determined by overlap in the feature of interest. As molecular datasets continue to grow and proliferate in the multi‐omics age, efficient morphological workflows become even more critical to allowing proportionally robust, complementary biological inferences grounded in phenotypic data.

## Graphical abstract

We present an efficient, broadly applicable, and open source approach to quantifying micron scale features using a machine learning pixel classification workflow. Using this method, we assess microtrichial variation in semiaquatic Ephydridae species and compare the results to manual assessment. Results are concordant and suggest a relationship between microtrichial length and number in the context of hydrophobicity.

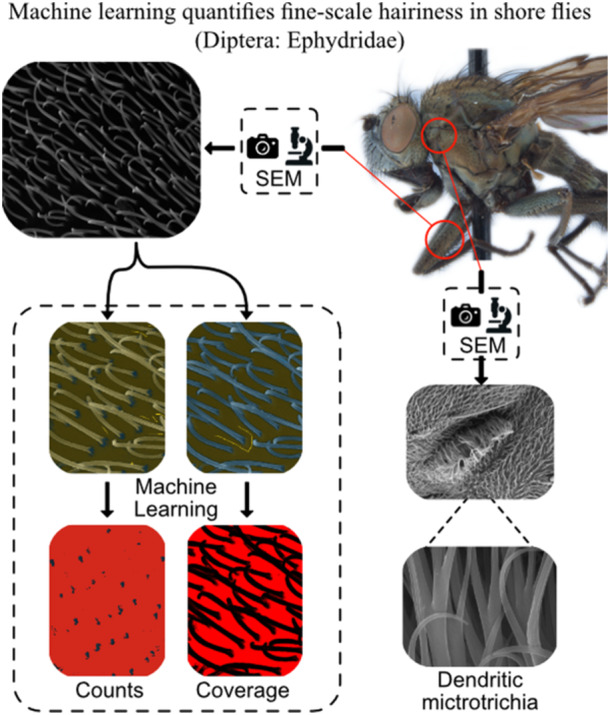

## 1 Introduction

Morphological data analysis is a cornerstone of many biological disciplines, whether to support taxonomy, functional aspects of ecology, or time‐calibrated phylogenies (Lee and Palci 2015). However, morphological study is limited by the time and training needed to code character states for large datasets, by biases in traditionally used characters, and by optical and practical limits of equipment (Wiens 2001; Weisenburger and Sandoghar 2015; Wipfler et al. 2016). Even if coded carefully, physical traits can be unequally informative for taxonomy, which seeks diagnostic uniformity, versus evolutionary studies that seek trait variation to determine biological relevance (Darwin 1871; Kimura 1977; Eyre‐Walker and Keightley 2007). These challenges can all be addressed through more efficient quantification of morphological character variation.

The use of artificial intelligence (AI) through machine learning to quantify morphological characters is rapidly increasing in popularity and application breadth (He et al. 2024). Once a workflow is defined, simple quantitative measurements can be reliably automated to reduce labor and expert training time in fields including taxonomic identification (Bhuiyan et al. 2022; Schneider et al. 2018), morphometrics (Bellin et al. 2022), video analysis of behaviour (Ratnayake et al. 2023) and medical diagnostics (Khalifa and Albadawy 2024). Here, we use pixel classification workflows in Ilastik (RRID:SCR_015246) to recognize morphological features by training a supervised machine learning AI employing a Random Forest model (Berg et al. 2019), and we compare its accuracy to manual methods. While other authors have produced related workflows to perform AI‐assisted, real‐time quantification of insect pollinator hairiness (Roquer‐Beni et al. 2020), we present a more accessible and widely applicable method using open source AI software and scanning electron microscopy (SEM). Using this method, we investigate the morphology of aquatic adaptations of insects with a specific focus on highly numerous, small‐scale, adaptive features.

Hydrophobicity and water repellence are at the core of insect aquatic adaptation, imparted by rough surface elements that maintain an unwetted state (Cassie and Baxter 1944; Law 2014). The degree of hydrophobicity depends on fluid variables affecting cohesion and surface tension, such as temperature or concentrations of salts and surfactants (Berthelot 1858; Pal 1950; Kunz et al. 2004; van Breugel and Dickinson 2017). However, the contacted surface structure and material are also critical. Insects achieve hydrophobicity through a combination of waxy cuticular secretions and densely‐packed setation (Maliński et al. 1986; Neumann and Woermann 2013; Botella‐Cruz et al. 2017). Hairs pin a stable or metastable point of interaction between air and water, maintaining surface‐attached bubbles during insect activity to prevent flooding of the spiracles or cuticle (Tesler et al. 2024). These hairs vary in morphology and arrangement to facilitate gas exchange across the bubble interface and create the conditions for water‐walking and droplet shedding interactions (Bush et al. 2008; Flynn and Bush 2008; Polet et al. 2015).

Setation in Diptera can be categorized into macrotrichia, which encompass larger setae and smaller setulae that insert through outer cuticle layers, and microtrichia, which are even smaller socketless projections of the outermost cuticle (McAlpine et al. 1981). Microtrichia can cover the entire body, making them a strong candidate for a primary hydrophobic roughness element. Their structure varies in the taxa investigated here, although typically appearing flattened with parallel longitudinal grooves. This contrasts with the cylindrical and helically grooved form of setae, which is conserved in the same taxa. Abundant microtrichia significantly increase the total body surface area and are so small that they require SEM or equivalent microscopy methods to resolve (Richards and Richards 1979). In Ephydridae, prior study including SEM imaging has been conducted, but focuses on larval adaptation (Wichard et al. 2002). Most insects have still not been examined using SEM (but see Espíndola Godoy et al. 2020; Kownacki et al. 2015; Laiolo et al. 2021; Xiang et al. 2016). Thus, quantification of SEM images of minute insects such as adult shore flies offers fertile ground for valuable discoveries on the contributions of morphology to properties like hydrophobicity.

The habitat associations of adult shore flies range from fully terrestrial to submerged aquatic feeding using surface‐attached bubbles (e.g. *Ephydra hians*) (Wirth 1971; Herbst and Bradley 1993; Foote 1995). This intriguing aquatic feeding behavior has long been observed by Indigenous groups and has more recently been investigated experimentally (van Breugel and Dickinson 2017; Toosarvandani 2020). Shore flies show wide environmental tolerance to habitats including saline lakes, hot springs, and petroleum pits (Mathis and Zatwarnicki 1995). Many live at high risk of water contact but must resist wetting to survive, as adult shore flies cannot actively swim, wetted flies cannot fly, and wetted spiracles impede respiration. Sex based differences in life histories (e.g. oviposition, nutritional requirements) may increase female risk (Ng et al. 2019). Since shore flies are densely covered in setae and microtrichia, our AI‐assisted workflow offers an efficient path from SEM images to quantitative data.

In light of successful applications in other domains and the potential of AI to expedite more extensive morphological analyses, we demonstrate that an AI‐assisted pipeline designed for numerous small‐scale features is comparable to manual measurement. Given the relevance of setation for environmentally specific aquatic adaptation, we expected commensurate variation in the extent and nature of microtrichia. Flies adapted to hot spring or saline conditions should be more hydrophobic, which should be reflected in microtrichial structure, length, density, or a combination thereof. Finally, we discuss the applicability of our pipeline and comment on measurement efficiency and fidelity in future implementations.

## 2 Materials and Methods

### 2.1 Included Species

We focused our analyses on three species of shore fly: *Paracoenia turbida* (Curran 1927), *Paracoenia bisetosa* (Coquillet 1902), and *Parydra aquila* (Fallén 1813) (Table 1). *Paracoenia turbida* is well adapted to hot spring systems, with abundant adults walking into or on water and saturated substrate (Collins 1977). *Paracoenia bisetosa* is also tightly associated with aquatic habitats and is widely distributed across typical wetland environments, but can be collected at both saline and hot spring sites (unpublished data, S. Abraham). *Parydra aquila* adults are widespread, are not known for their environmental tolerance, and are aquatic to a lesser extent as neuston components compared to *Paracoenia* species that can partially submerge. All three quantitatively analyzed species have similar body size. We also included *Mosillus bidentatus* (Cresson 1926) and *Ephydra hians* Say 1830 in the initial qualitative assessment to better demonstrate variability across habitat associations (Table 1). *Mosillus bidentatus* is slightly smaller and associated with the water's edge, but occurs neither atop the surface nor submerged. By contrast, *Ephydra hians* is larger and strongly water‐associated (Herbst and Bradley 1993).

**Table 1:** Collection information and specimen data for all included material.

Species	Locality	Prov./State, Country	Year	# Coll., Sex	Mean Body Length (μm), without head (Figure S1)
*Paracoenia bisetosa*	Buffalo Lake	AB, CAN	2022	4 F, 4 M	3860.8 (95% CI ± 321.3)
*Paracoenia turbida*	Rabbit Creek, Yellowstone Park	WY, USA	2023	4 F, 4 M	3231.2 (95% CI ± 187.0)
*Parydra aquila*	Itaska Beach, Pigeon Lake Red Deer Riverbed, Spruce View	AB, CAN AB, CAN	2023 2023	1 F, 2 M 3 F, 2 M	3551.0 (95% CI ± 127.5)
*Mosillus bidentatus*	Red Deer Riverbed, Spruce View	AB, CAN	2022	1 F	2909.96
*Ephydra hians*	Olivia Lake	AB, CAN	2023	1 F	4536.03

### 2.2 Specimen Preparation and Imaging

Specimens were netted or aspirated, then stored in either 70% or 95% ethanol. Specimens were visually sexed based on terminal abdomen shape and identified following McAlpine et al. (1981) and Mathis (1975) (Table 1). Yellowstone National Park specimens were collected under permit YELL‐2023‐SCI‐8100 held by Dr. R.K. Peterson, Montana State University.

We prepared and imaged all specimens at the University of Alberta's Advanced Microscopy Facility. Flies were serially diluted from ethanol into distilled water. To remove surface contaminants, we ultrasonically cleaned each fly in 15 mL Corning centrifuge tubes of warm distilled water and mild detergent for 40 min at 117 V/60 Hz using a Branson 2510 sonicator. We rinsed then fixed specimens in Karnovsky fixative for at least 24 h before dissection. A modified hexamethyldisilazane (HMDS) protocol (modified from Bozzola and Russell 1992 to exclude OsO_4_ steps) was used to dehydrate dissected tissue before SEM stub mounting. Specimens were sputter‐coated with gold‐palladium before imaging.

Anatomical locations were selected based on their hypothesized biological relevance for aquatic adaptations, such as producing and maintaining surface attached bubbles, and the ease of imaging consistency. We then filtered methodologically incompatible characters such as the lack of flat surface and nonuniform microtrichial angles found around prothoracic shore fly spiracles, or those with specimen damage.

**Figure 1:**
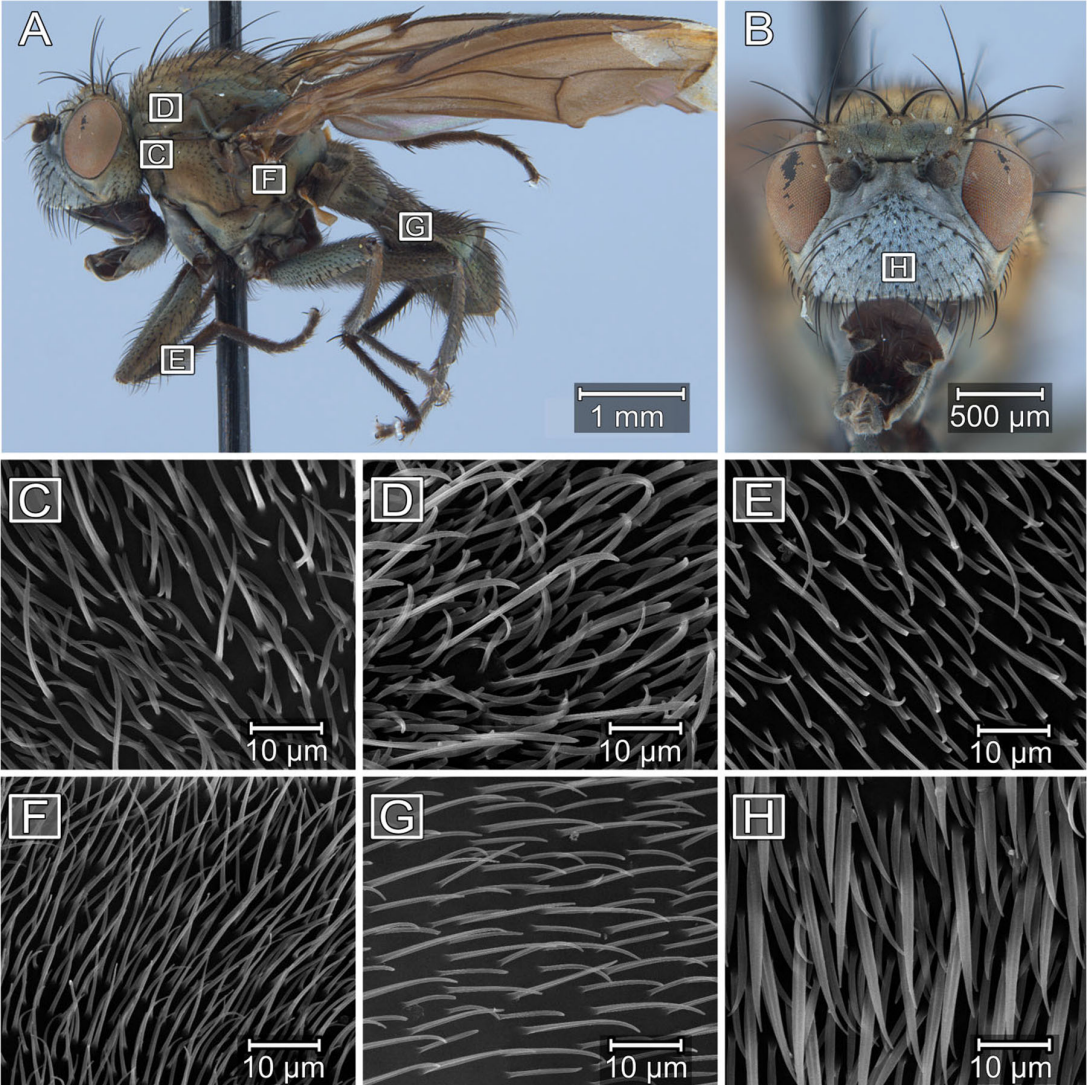
Shore fly anatomy, with SEM microtrichial images used for quantitative analysis. Lateral view (A) and anterior view (B) of ***Paracoenia bisetosa***. 5000× magnified views of anatomical locations imaged in this study and summarized in Table S1: Dorsal to the anterior thoracic spiracle **AS** (C), Post‐pronotal lobe **PPRN** (D), Lateral prothoracic femur **FE** (E), Ventral to the posterior to the thoracic spiracle **PS** (F), Abdominal sclerite 3, **A3** (G). Face, **FA** (H).

Specific imaging frames were standardized within anatomical locations by mounting specimens in fixed orientations, locating the region of interest, then visually searching for an image frame that minimized visible damage or surface contaminants (Figure 1, Table S1).

We visualized microtrichia using a ZEISS EVO 10 scanning electron microscope at a 20 kV working acceleration voltage to image at 10000×, 5000×, and 2500× magnification for quantitative analyses and up to 50000× magnification for qualitative descriptions. We oriented each specimen consistently on each mount to standardize the beam exposure (Figure S1), and adjusted the brightness and contrast during imaging then normalized both with Affinity Photo v1.10.6.1665 (Serif Europe Ltd. 2025) (RRID:SCR_016951) after imaging.

### 2.3 Image Analysis

We used two methods each to measure microtrichial lengths and densities. For lengths, we first overlaid a 4 × 3 grid and randomly selected 3 quadrats per anatomical location per specimen for four males and four females of *Parydra aquila, Paracoenia bisetosa* and *Paracoenia turbida*. We then measured the lengths of three sampled microtrichia per quadrat with the segmented line tool in Fiji (Schindelin et al. 2012) (RRID:SCR_002285) for a subtotal of 54 measurements per specimen, totalling 432 measurements per species. All imaged anatomical locations were included because microtrichial density was sufficient to sample comparably from each quadrat. For the second length measurement, we took a subset of 9 images of female specimens for all three species and comprehensively measured all complete microtrichia present in each image. Here, PPRN and PS were excluded due to within‐specimen irregularity of microtrichial angles, and FA was excluded due to morphological comparability of individual microtrichia between *Parydra* and *Paracoenia* species.

**Figure 2:**
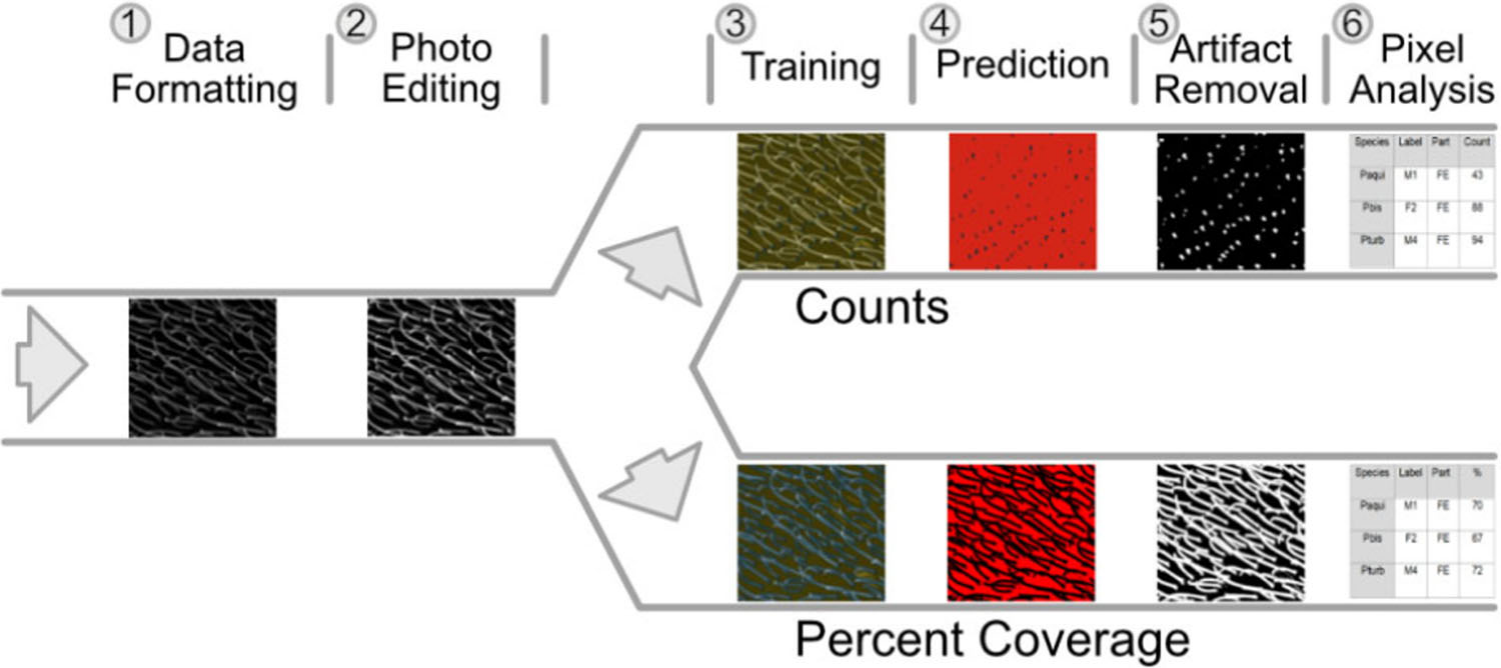
Ilastik ‐ Fiji AI‐assisted workflow to estimate density of microtrichia from scanning electron micrographs. Images were grouped by general similarity for independent prediction runs (1) then brightness and contrast were normalized in Affinity Photo (2). Grouped images were interactively trained in Ilastik (3) to produce prediction and segmentation maps (4). Maps of count data were eroded and despeckled in Fiji, while maps of coverage data were only despeckled (5). Finally, pixel clusters meeting the minimum threshold size were enumerated for count data, while the percentage of pixels classified as microtrichia was calculated for coverage data (6).

We measured density first with manual and AI‐assisted counts of microtrichia and then by calculating the percent hair coverage by AI pixel classification. Counts were made manually in two passes: First, all microtrichia with both the base and tip visible, then second, all visible uncounted bases to pre‐empt object classification issues in recognizing entire microtrichia. AI‐assisted counts and all percent coverage measurements were conducted with pixel classification workflows in Ilastik v1.4.0. For AI‐assisted counts, we grouped images by magnification and general similarity, then trained the pixel classifier to distinguish microtrichial bases from cuticle, setae, and surface contaminants (Figure 2). We interactively trained the model by classifying image elements, then updating predictions until prediction and uncertainty maps converged and passed a visual comparative inspection of a subset of the corresponding micrographs (Figure S2). We ran each trained group independently to create prediction maps of bases for each anatomical location of each species. Exported maps were eroded to split connected bases, despeckled to remove erroneously labelled small pixel clusters, and the resultant particles were counted in Fiji. The three included anatomical locations in count data (A3, FA, and FE) were the most informative due to their uniform substrate and microtrichial angles across all species. Density approximations by percent coverage used a similar pixel classification workflow to discriminate between microtrichia and other elements (Figure 2). The AI was trained analogously to count data, but instead compared pixels classed as microtrichia versus background in Fiji and without erosion. All imaged anatomical locations were included in coverage data.

We chose Ilastik v1.4.0 for its high input flexibility and range of both object and pixel classification systems. It accepts stacked or single images, receives ongoing support, and has a direct plugin for Fiji (ImageJ), while featuring a larger suite of native functions than more specialized software. We used the ggstatsplot package with non‐parametric settings in R v4.3.2 because datasets are small and Shapiro‐Wilk tests determined that count and length datasets were non‐normal (AI count: W = 0.953, *p* = 0.012, manual count: *W* = 0.953, *p* = 0.011, length: *W* = 0.721, *p* = 2.2e^−16^), though coverage data passed the Shapiro‐Wilk test weakly (*W* = 0.982, *p* = 0.07) (Shapiro and Wilk 1965; Patil 2021; Vrbin 2022). Three‐group comparisons were analyzed with Kruskal–Wallis one‐way ANOVAs, while two group comparisons were analyzed with Mann‐Whitney *U* tests (Kruskal and Wallis 1952; Mann and Whitney 1947). Dunn's test of multiple comparisons was used post hoc, and Bonferroni's adjustment was applied (Dunn 1961). The R packages ggstatsplot and ggplot2 were used to generate initial figures which were edited in Affinity Photo v1.10.6.1665 (SerifEurope Ltd. 2025) (Wickham 2009; Patil 2021; R Core Team 2024).

## 3 Results

**Figure 3:**
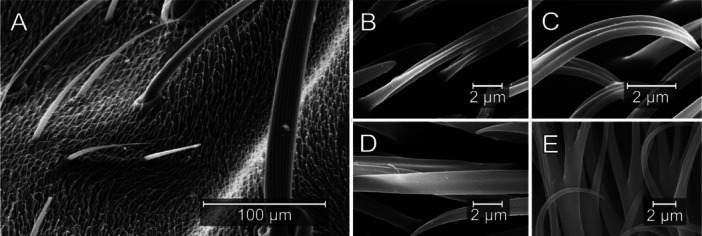
Types of setation on shore flies. *Paracoenia bisetosa* post‐pronotal lobe (PPRN) with visible bristles, setulae, and microtrichia (A) and a selection of microtrichial structures found on the body (B–E). PPRN, showing “featherlike” and “fingerlike” structures (B, C); FA, showing “bladelike” structure (D); AS, showing low texture and dendritic structure (E).

**Figure 4:**
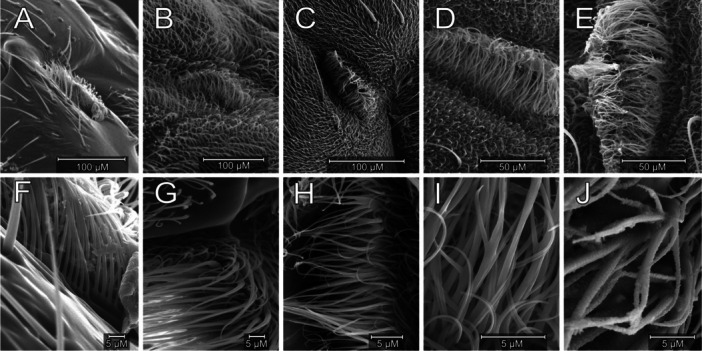
Variation in anterior thoracic spiracle (AS) microtrichia. Upper and lower rows represent paired images of the same species at lower (450×) and higher (10,000–20,000×) magnification, showing uniform, organized microtrichia (A, F—*Mosillus bidentatus*), nonuniform, organized microtrichia (B, G—*Parydra aquila*), and varying degrees of unorganized, nonuniform dendritic microtrichia (C, H—*Paracoenia bisetosa* and D, I—*Paracoenia turbida*). *Ephydra hians* (E, J) shows the most highly dendritic, least uniform microtrichia. Surface texture in J persisted after ultrasonic cleaning.

Microtrichial shape and structure varied qualitatively across taxa and anatomical locations and consistently showed longitudinal grooves, in contrast to conserved helical sculpturing on larger setae (Figure 3). Spiracular microtrichia had variable coverage patterns ranging from uniform and sparse to unstructured and dense. Dendritic microtrichia that share a single base and branch out apically into multiple tips on prothoracic spiracles appeared in two groups: the salt lake specialist *Ephydra hians* and both *Paracoenia* species (hot spring specialist *Paracoenia turbida* and habitat generalist *Paracoenia bisetosa)* (Figure 4).

**Figure 5:**
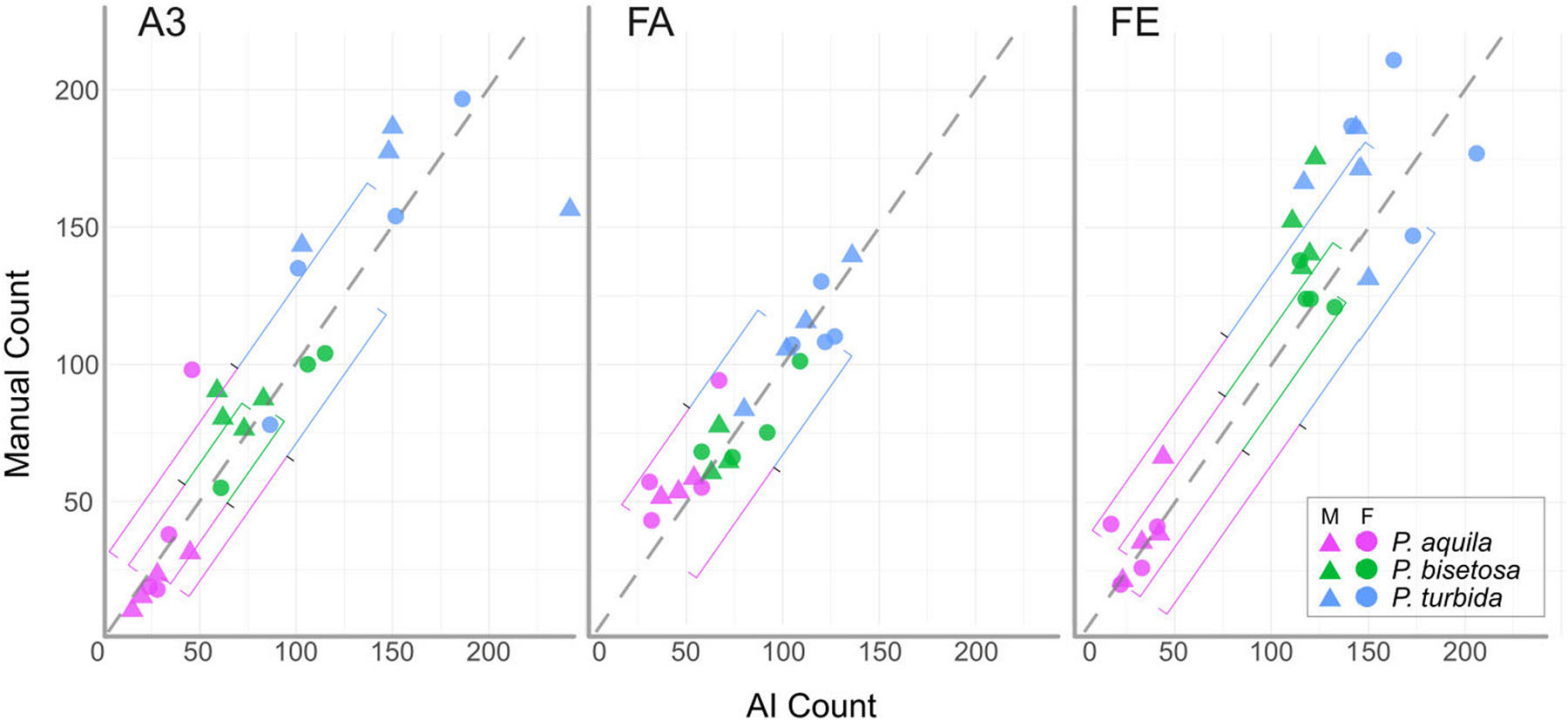
AI‐assisted versus manual counts of microtrichial bases in micrographs at 5000x magnification. Each symbol represents one micrograph. Species are denoted by color, sex is denoted by shape. Each plot represents one of the three most informative anatomical locations assessed (A3, FA, and FE). Dashed line indicates equal AI and manual counts. Coloured brackets denote significant pairwise differences for manual counts (above dashed line) and AI counts (below dashed line), with endpoints of brackets indicating means for sets of counts.

**Figure 6:**
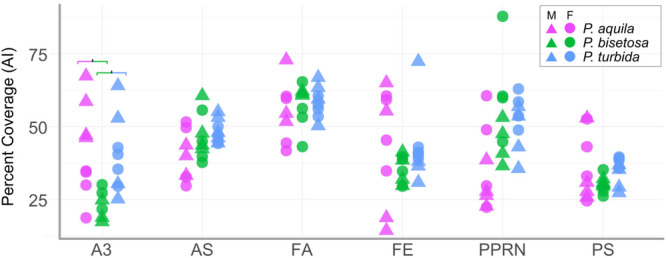
Variation in microtrichial density measured by classifying pixels in 5000× SEM images as either hair or background cuticle. Each symbol represents one micrograph. Species are denoted by color, sex is denoted by shape. The Y axis denotes the percentage of pixels classified as microtrichia in Ilastik. The X axis denotes anatomical locations, with abbreviations as defined in Figure 1, Table S1. Coloured brackets for A3 denote significant pairwise differences.

Quantitative analysis focussed on four male and four female specimens of *Paydra aquila, Paracoenia bisetosa*, and *Paracoenia turbida*. For the assessment of AI versus manual counts (Figure 5), 5935 microtrichia were counted manually and 6209 microtrichia were counted by AI, with both count methods using the same set of 72 images. For percent coverage (Figure 6), the same data set plus 70 additional images were used, for a total of 142. For length distributions (Figure S3), a total of 956 microtrichia across 9 images were manually measured, with 3 for each single female of a species. For detection of potential sexual dimorphism in microtrichial lengths (Figure S4, a total of 1278 microtrichia across 142 images were manually measured.

We did not detect intraspecific sexual dimorphism in microtrichial lengths (Figure S4, AI‐assisted counts (**AI**: *Paydra aquila* W_Mann–Whitney_ = 64.50, *p* = 0.95, *Paracoenia bisetosa* W_Mann–Whitney_ = 73.00, *p* = 0.69, *Paracoenia turbida* W_Mann–Whitney_ = 73.00, *p* = 0.64) or manual counts (**Manual**: *Paydra aquila* W_Mann–Whitney_ = 81.50, *p* = 0.58, *Paracoenia bisetosa* W_Mann–Whitney_ = 56.00, *p* = 0.79, *Paracoenia turbida* W_Mann–Whitney_ = 51.00, *p* = 0.57) (Figure 5).

Ilastik microtrichial counts were not significantly different from manual counts at the same anatomical location for eight of the nine pairwise comparisons. For *Parydra aquila*, no differences were significant (**A3:** W_Mann_ = 39.50, *p* = 0.77, **FA**: W_Mann_ = 18.00, *p* = 0.44; **FE**, W_Mann_ = 28.50, *p* = 0.75). For *Paracoenia bisetosa*, the difference between AI and manual counts was significant on femora (**A3:** W_Mann_ = 28.00, *p* = 1.00, **FA:** W_Mann_ = 31.50, *p* = 0.41 **FE:** W_Mann_ = 4.00, *p* = 0.00385). For *Paracoenia turbida*, no differences were significant (**A3:** W_Mann_ = 8.00, *p* = 0.07, **FA:** W_Mann_ = 38.00, *p* = 0.56, **FE:** W_Mann_ = 18.00, *p* = 0.16). Ilastik and manual counts tended to deviate more at higher total numbers of microtrichia (Figure 5).

Microtrichial counts were fewest in *Parydra*, followed by *Paracoenia bisetosa*. *Paracoenia turbida* had the most microtrichia per image for all regions tested with complete datasets (Figure 5). There were significant differences between species within all anatomical locations for both AI counts (**A3:** χ^2^_Kruskal–Wallis_ = 15.66, *p* = 3.98e^−4^, CI_95%_ [0.69, 1.00], **FA:** χ^2^_Kruskal–Wallis_ = 16.36, *p* = 2.81e^−4^, CI_95%_ [0.67, 1.00], **FE:** χ^2^_Kruskal–Wallis_ = 19.01, *p* = 7.44e^−5^, CI_95%_ [0.74, 1.00]) and manual counts (**A3:** χ^2^_Kruskal–Wallis_ = 17.15, *p* = 1.89e^−4^, CI_95%_ [0.76, 1.00], **FA:** χ^2^_Kruskal–Wallis_ = 15.90, *p* = 3.52e^−4^, CI_95%_ [0.76, 1.00], **FE:** χ^2^_Kruskal–Wallis_ = 18.02, *p* = 1.22e^−4^, CI_95%_ [0.69, 1.00]).

The significance of differences in microtrichial counts among species within anatomical locations was shared by AI and manual count results (Figure 5). Counts for all three locations assessed were significantly different between *Paydra aquila* and *Paracoenia turbida* (AI counts: **A3**: *p* = 6.33e^−4^, **FA:** *p* = 1.60e^−4^, **FE:** *p* = 4.09e^−5^) (Manual counts: **A3**: *p* = 1.95e^−4^, **FA:** *p* = 2.42e^−4^, **FE:** *p* = 7.69e^−5^), but were only significant between *Paydra aquila* and *Paracoenia bisetosa* on the abdomen and femora (AI counts: **A3**: *p* = 7.95e^−4^, **FE:** *p* = 0.04) (Manual counts: **A3**: *p* = 0.02, **FE:** *p* = 0.03). Microtrichial counts were not significantly different between *Paracoenia bisetosa* and *Paracoenia turbida*.

Percent microtrichial coverage showed a total combined range of 14.7%–87.9% and few significant differences between species (Figure 6). The facial location showed the highest average coverage (41.8%–73.3%), and the posterior spiracle showed the lowest (24.5%–53.4%). Other regions generally range between 41.7% and 73% coverage, except for A3 on *Paracoenia bisetosa*. *Parydra aquila* showed the most variation in coverage between individuals of the same species. There were only two statistically significant differences between species within an anatomical location, both on the abdomen (**A3:** χ^2^_Kruskal–Wallis_ = 8.28, *p* = 0.016, CI_95%_ [0.22, 1.00]): between *Paydra aquila* and *Paracoenia bisetosa* (*p* = 0.028) and between *Paydra aquila* and *Paracoenia turbida* (*p* = 0.038). The postpronotal lobe showed overall significant variation (**PPRN:** χ^2^_Kruskal–Wallis_ = 6.31, *p* = 0.043 CI_95%_ [0.5, 1.00]) but pairwise comparisons were insignificant.

## 4 Discussion

### 4.1 Biological Findings

We successfully applied a machine‐learning method to quantify and compare fine‐scale hairiness based on two metrics: microtrichial base counts and coverage. Our trial using three species highlights the importance of distinguishing counts (numbers of microtrichial bases) from coverage (proportion of surface covered). There were more significant differences between species in the absolute number of microtrichia than there were in percent coverage, despite the similarity among these species in body length (Table 1). This suggests that resistance to wetting is not strictly due to coverage, which aligns with experimental data on wetting and the roughness element of hydrophobicity (van Breugel and Dickinson 2017; Flynn and Bush 2008).

Despite significant differences in microtrichial number and length between species, differences between males and females of the same species were not evident, even with differences in behavior such as some female shore flies ovipositing on or below the water's surface (Figure 5, S4) (Herbst 1988). This allowed larger combined‐sex sample sizes in later analyses and also suggests that different life history characteristics and environmental tolerances of the sexes of these taxa may not be significantly associated with different microtrichial patterns.

The combined effects of microtrichial length and number may account for why coverage generally did not differ between species while counts did. The most terrestrial species, *Parydra aquila*, had the fewest hairs by count but the longest microtrichia in almost every anatomical location (Figure 5, S4). Conversely, the most aquatic species, *Paracoenia turbida*, had the highest average number of microtrichia in all measured areas and the shortest in some regions. *Paracoenia bisetosa* was intermediate for count and mixed for length and is also ecologically intermediate. Since the size and spacing of surface roughness elements affects inherent hydrophobicity (Du et al. 2022), this relationship may be biologically relevant.

Our observations on the two species we used to help bracket and define characters together with the three focal species demonstrated other variables of interest in the organization and surface structure of the microtrichia themselves. Roughness in relation to wetting resistance can be expressed hierarchically (Yang et al. 2006). In addition to large setae, most Diptera possess assemblies of setulae and microtrichia. Structure at all scales may contribute to the roughness required to retain an air layer between insects and water. We observed a variety of structural differences and provide the first report of dendritic spiracular microtrichia in *Paracoenia* and *Ephydra*, but further analysis is beyond the scope of this study.

### 4.2 Effectiveness of Machine Learning Application

Our approach is particularly suitable for small insects and micron‐scale features like microtrichia. Other methodologies that have been used to compare hairiness in insects (Roquer‐Beni et al. 2020) are not as well suited to the scale and quantitative needs of our study system. These methods are best applied to larger specimens with long, dense setae like bumblebees and moths, and require specimens to be shaved to reveal setal insertion points for analysis via real‐time photomicrography analysis equipment.

We anticipate that our method will have increased efficiency as datasets grow. AI‐assisted quantification was substantially more efficient than manual counting, while manual assessment of percent coverage is impractical. For our data set, manual microtrichial counts required an estimated 2.5 min per image, with limited efficiency gained through practice. In contrast, a defined Ilastik workflow and a preset Fiji macro can generate similar data at an estimated 1.0 min per image for a data set, and this can be expected to decrease as the number of input images increases. In addition, semi‐automated workflows should reduce errors and user fatigue.

### 4.3 Sources of Manual Measurement Error

Inherent measurement bias and error in morphological data is often under‐examined, and has recently been addressed within a formal framework (Collyer and Adams 2024). Our study addresses two major elements of measurement bias: 2D image analysis bias and AI training bias, and we identified and minimized sources of error when possible (Borges et al. 2020; Koçak et al. 2025). Within anatomical locations, microtrichial curve skews length measurements due to perspective error. To address this and downstream comparability, we maximized imaging angle similarity to reduce the proportion of random error (Figure S1). Alternatively, imaging parallel to the cuticle and perpendicular to microtrichia would increase accuracy but drastically reduce sampling efficiency. Additionally, *Z*‐stacked images from this alternative angle increase the number of microtrichia per image but greatly increase overlap.

The overall effect of high overlap is an upper boundary for accurate density estimation. Very high densities produce overlap that blocks some fraction of microtrichia from view. Thus, single image AI workflows and manual methods will both involve systemic undercounting. *Z*‐stacked image sets could mitigate this since uncompiled image stacks are accepted inputs in many Ilastik workflows.

The de‐speckling and erosion steps of our workflow may also introduce over‐ or undercounting error. The minimum size threshold for a pixel object is user defined and varies with magnification and feature size. A low threshold can split bases into multiple objects, and a high threshold merges adjacent bases. This can be solved by iteratively testing threshold values until a small sample of manual data converges with AI outputs for a given feature size.

### 4.4 Sources of AI Measurement Error

Ilastik interactively provides users with visual feedback of pixel class predictions and degree of uncertainty. After training, the highest degree of uncertainty was found in microtrichial base data at the boundaries between the edges of microtrichial bases and surrounding cuticle, and the apical tips of microtrichia (Figure S2). However, because downstream analysis of these data depended on the presence of pixel clusters above a manually set size threshold independent of uncertainty, we expect the impact to be negligible.

The greyscale nature of SEM and shape of microtrichia combined with significant overlap reduced the available parameters for object‐based image classification workflows to distinguish discrete microtrichial objects to the extent that our AI classifier could not perform reliably. However, the characteristic shading patterns at the bases of microtrichia provided a consistent parameter set for the pixel‐based workflows which were ultimately used (Figure 2). This type of approach was accurate in our study but could theoretically introduce error if manual assessment inferred the presence of fully or partially concealed features invisible to our AI‐based workflow (Figure 5).

Ilastik was developed for cell biology and excels at distinguishing less linear structures than microtrichia. One positive outcome of our pixel‐classification workflow was that damaged or missing microtrichia remained recognizable as the same pixel class. In other applications where damaged features are not recognizable as the same pixel class, classifying damaged features separately and summing the two layers would solve this. This methodology can be applied broadly across taxa if the features of interest are consistent in appearance.

Lighting and contrast normalisation can greatly improve AI classifier consistency (Berg et al. 2019). SEM relies on balanced interactions between the beam, the backscatter sensor, and sample discharge (Bozzola and Russell 1992). High points such as setae may retain charge, resulting in bright washout that is difficult to mitigate in post‐processing and is best addressed before or during imaging. Datasets based on fluorescent or light microscopy that includes hue variables should be useful for quantifying more complex features.

## 5 Conclusion

This study presents a flexible, open‐source and efficient approach for quantitative assessment of morphological features at scales necessitating scanning electron microscopy. We also suggest solutions to potential limitations in future applications. For semiaquatic adult shore flies, we show substantial variation in previously unassessed microtrichial patterns, including between congeners with overlapping ecological niches, and suggest that trade‐offs between the number and length of microtrichia are likely to be adaptive in the context of hydrophobicity.

## Author Contributions

**Shawn M. Abraham:** conceptualization (lead), investigation (equal), methodology (equal), data curation (equal), formal analysis (lead), visualization (equal), supervision (equal), writing – original draft (lead), writing – review and editing (equal). **Marcos Rodriguez:** investigation (equal), visualization (equal), methodology (equal), data curation (equal). **Victoria Hristova:** investigation (equal), visualization (equal), data curation (equal). **Felix A. H. Sperling:** project administration (lead), funding acquisition (lead), resources (lead), conceptualization (equal), supervision (equal), writing – review and editing (equal).

## Ethics Statement

The authors have nothing to report.

## Conflicts of Interest

The authors declare no conflicts of interest.

## Supporting information


**Supplemental Figure 1:** Anatomical arrangement of specimens on SEM stubs. **Supplemental Table 1:** Standardization of anatomical locations used for SEM imaging. **Supplemental Figure 2:** Image segmentation process of SEM microtrichial images for counts and coverage in Ilastik. **Supplemental Figure 3:** Violin plots of microtrichial length distributions for the three most comparable anatomical locations among *Parydra aquila, Paracoenia bisetosa,* and *Paracoenia turbida*. **Supplemental Figure 4:** Sex‐associated microtrichial length: body length ratios for *Parydra aquila, Paracoenia bisetosa*, and *Paracoenia turbida*.

## Data Availability

The image data set used in the production of this study is freely available in a digital repository doi: 10.5281/zenodo.16548030. Specimens mounted for SEM imaging will be housed in the E.H Strickland Entomological Museum at the University of Alberta in Edmonton, Alberta, Canada.
