# Supplementary material for: Machine Learning Quantifies Fine‐Scale Hairiness in Shore Flies (Diptera: Ephydridae)
Source: J Morphol. 2025 Oct 14;286(10):e70096. doi: 10.1002/jmor.70096 (PMC12519760; doi:10.1002/jmor.70096)
Supplement: Supplementary file 1 — Supplemental Figure 1: Anatomical arrangement of specimens on SEM stubs. Supplemental Table 1: Standardization of anatomical locations used for SEM imaging. Supplemental Figure 2: Image segmentation process of SEM microtrichial images for counts and coverage in Ilastik. Supplemental Figure 3: Violin plots of microtrichial length distributions for the three most comparable anatomical locations among Parydra aquila, Paracoenia bisetosa, and Paracoenia turbida. Supplemental Figure 4: Sex‐associated microtrichial length: body length ratios for Parydra aquila, Paracoenia bisetosa, and Paracoenia turbida. [file JMOR-286-e70096-s001.docx]

**Supplemental Material**


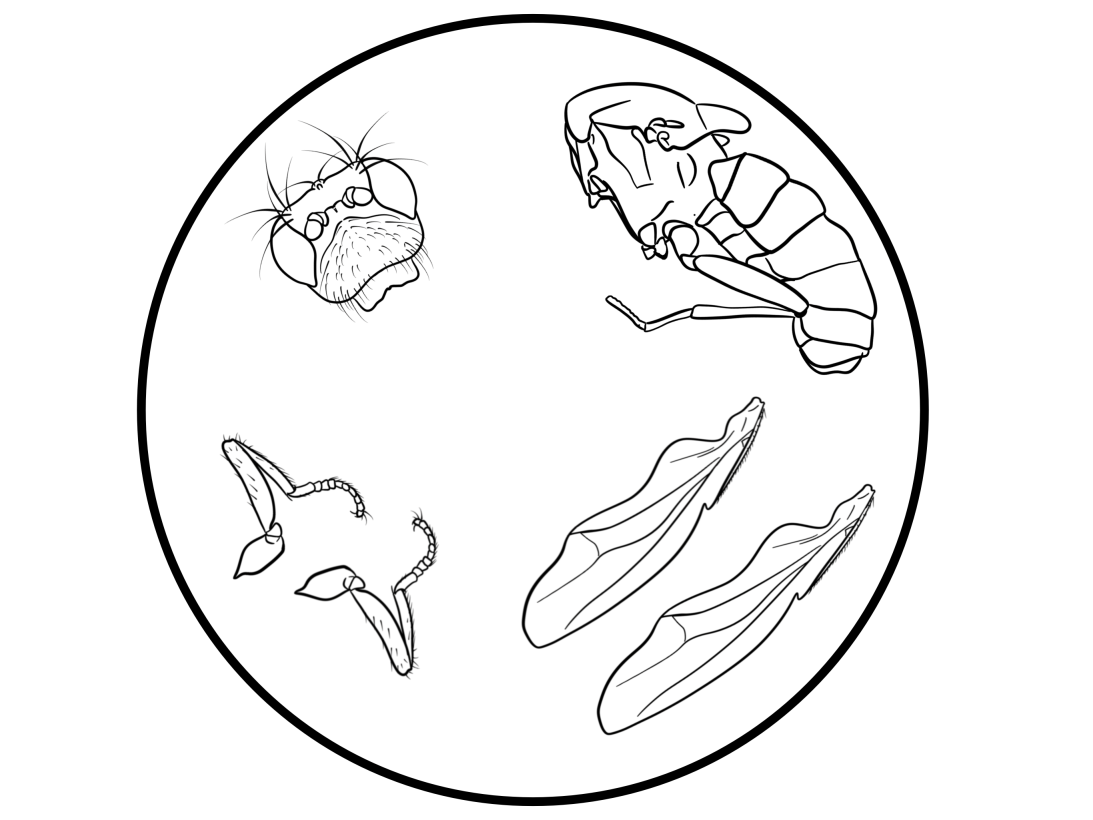


**Supplemental Figure 1. Anatomical arrangement of specimens on SEM stubs.** Specimen parts were placed in radial symmetry in the vacuum chamber to ensure even beam coverage.

**Supplemental Table 1: Standardization of anatomical locations used for SEM imaging**

| **Anatomical Location (Abbreviation)** | **Specific region of imaging** |
| --- | --- |
| Facial region (FA) | Center of facial region, typically laterally adjacent to the centerline. |
| Abdomen (A3) | Just anterior to the posterior edge of A3 sclerite |
| Anterior Thoracic Spiracle (AS) | Flat area just dorsal and anterior to the spiracle |
| Posterior Thoracic Spiracle (PS) | Flat area just ventral to the spiracle, where two hair patterns meet. Image centered halfway between them. |
| Postpronotal lobe (PPRN) | Triangulated by forming a right triangle with two bristles on the postpronotum |
| Femur (FE) | Prothoracic femur, proximal end of dorsolateral surface |


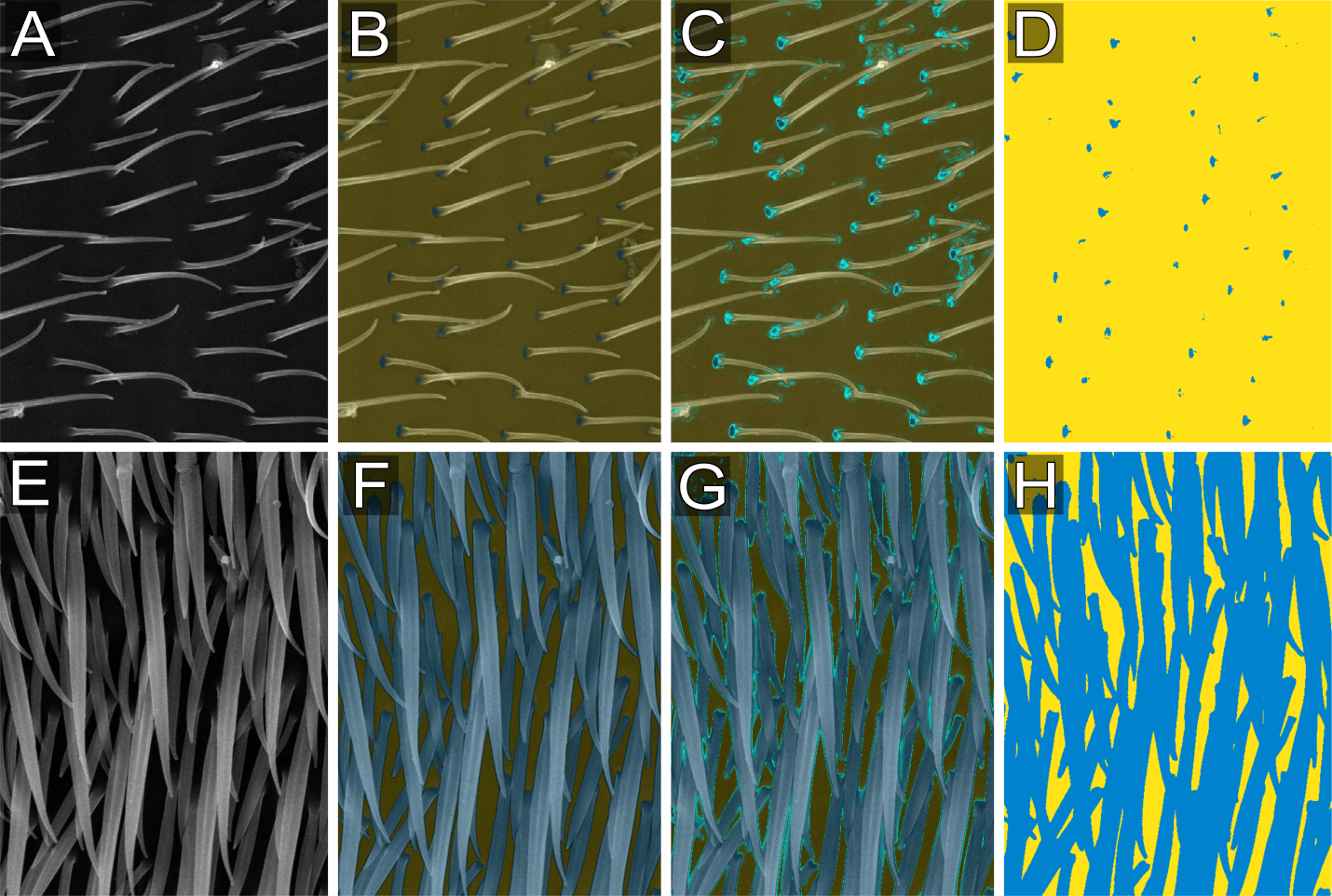


**Supplemental Figure 2:** **Image segmentation process of SEM microtrichial images for counts and coverage in Ilastik.** Images of *Paracoenia bisetosa* abdomen (A3), trained to count microtrichia (A-D), and *Paracoenia bisetosa* facial region (FA), trained to calculate microtrichial coverage (E-H) are shown from left to right in order of generation. Raw images (A, E) are input into Ilastik in groups based on body region, then prediction maps (B, F) are generated through training and prediction. Uncertainty maps (C, G) showing the location and degree of uncertainty in cyan are produced and checked before exporting segmented images (D, H) for downstream analysis.


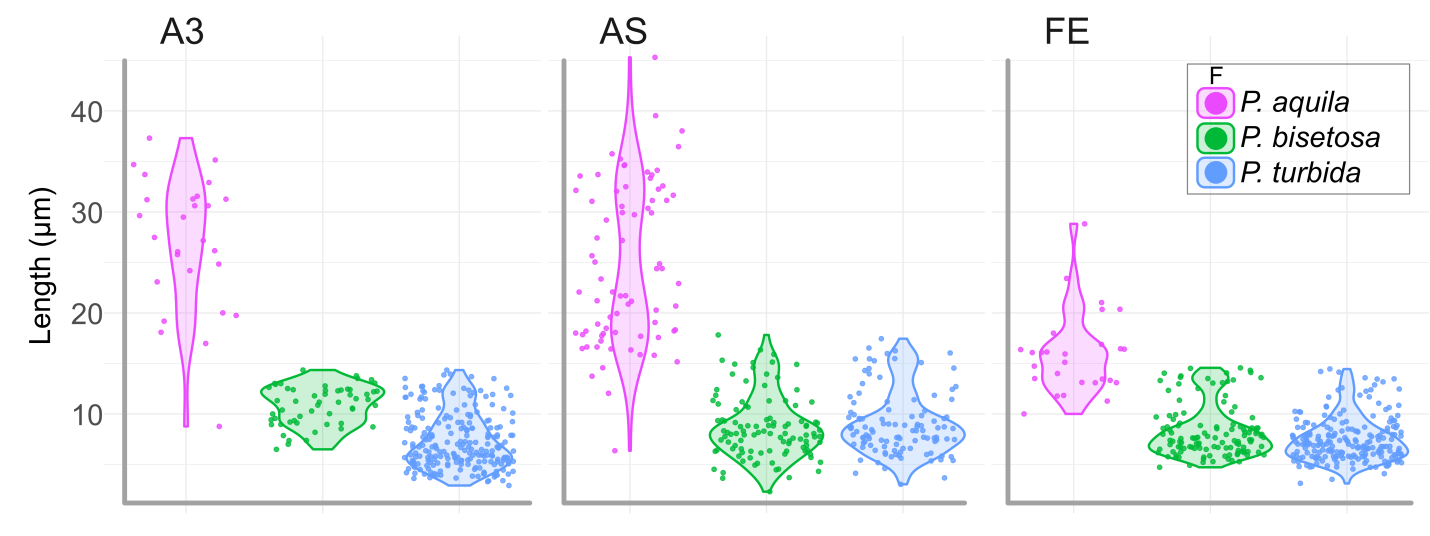


**Supplemental Figure 3 Violin plots of microtrichial length distributions for the three most comparable anatomical locations among *Parydra aquila, Paracoenia bisetosa,* and *Paracoenia turbida*.** Each dot represents one microtrichium on the abdomen (A3), anterior thoracic spiracle (AS), and femur (FE). All microtrichia in a single image of a single female were measured for each species. Total n = 953 microtrichia (*Py. aquila* = 129, *Pc. bisetosa* = 285, *Pc. turbida* = 541).


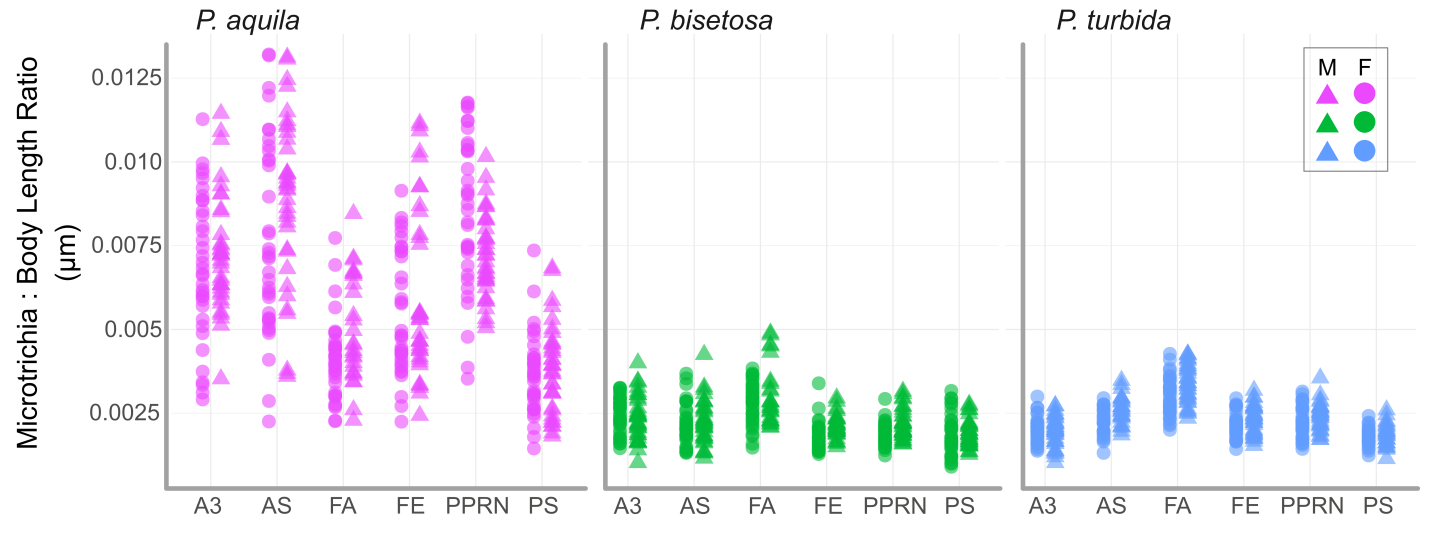


**Supplemental Figure 4: Sex-associated microtrichial length: body length ratios for *Parydra aquila, Paracoenia bisetosa*, and *Paracoenia turbida*.** Each symbol represents one microtrichium. Nine microtrichia were sampled per each of 6 anatomical locations for each of 8 specimens per species. Total n = 1296 microtrichia. Ratios were obtained by dividing measured microtrichia by the body length of the associated specimen as in Table S1. Species are denoted by color, sex by shape. Anatomical abbreviations are as in Figure 1, Table S1.

**Graphical Abstract / TOC Text**


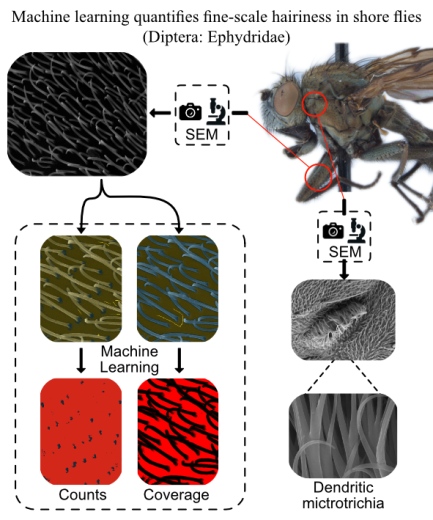


Machine learning quantifies fine-scale hairiness in shore flies (Diptera: Ephydridae)

Authors: Abraham S.M.*^1^, Rodriguez M.^1^, Hristova V.^1^, Sperling F.A.H.^1^

We present an efficient, broadly applicable, and open source approach to quantifying micron scale features using a machine learning pixel classification workflow. Using this method, we assess microtrichial variation in semiaquatic Ephydridae species and compare the results to manual assessment. Results are concordant and suggest a relationship between microtrichial length and number in the context of hydrophobicity.
